# A comparison of the conditional inference survival forest model to random survival forests based on a simulation study as well as on two applications with time-to-event data

**DOI:** 10.1186/s12874-017-0383-8

**Published:** 2017-07-28

**Authors:** Justine B. Nasejje, Henry Mwambi, Keertan Dheda, Maia Lesosky

**Affiliations:** 1 School of Statistics, Mathematics and Computer Science, University of Kwazulu-Natal, Pietermaritzburg, South Africa; 2 Division of Pulmonology and UCT Lung Institute, Department of Medicine, University of Cape Town, Cape Town, South Africa; 3 Division of Epidemiology and Biostatistics, School of Public Health and Family Medicine, University of Cape Town, Cape Town, South Africa

**Keywords:** Survival analysis, Split-points, Survival trees, Random survival forests, Conditional inference forests

## Abstract

**Background:**

Random survival forest (RSF) models have been identified as alternative methods to the Cox proportional hazards model in analysing time-to-event data. These methods, however, have been criticised for the bias that results from favouring covariates with many split-points and hence conditional inference forests for time-to-event data have been suggested. Conditional inference forests (CIF) are known to correct the bias in RSF models by separating the procedure for the best covariate to split on from that of the best split point search for the selected covariate.

**Methods:**

In this study, we compare the random survival forest model to the conditional inference model (CIF) using twenty-two simulated time-to-event datasets. We also analysed two real time-to-event datasets. The first dataset is based on the survival of children under-five years of age in Uganda and it consists of categorical covariates with most of them having more than two levels (many split-points). The second dataset is based on the survival of patients with extremely drug resistant tuberculosis (XDR TB) which consists of mainly categorical covariates with two levels (few split-points).

**Results:**

The study findings indicate that the conditional inference forest model is superior to random survival forest models in analysing time-to-event data that consists of covariates with many split-points based on the values of the bootstrap cross-validated estimates for integrated Brier scores. However, conditional inference forests perform comparably similar to random survival forests models in analysing time-to-event data consisting of covariates with fewer split-points.

**Conclusion:**

Although survival forests are promising methods in analysing time-to-event data, it is important to identify the best forest model for analysis based on the nature of covariates of the dataset in question.

## Background

The Cox-proportional hazards model [1] is a popular choice for analysis of right censored time-to-event data. The model is convenient for its flexibility and simplicity, however, it has been criticised for its restrictive proportional hazards (PH) assumption [2–4] which is often violated. A number of extensions to the Cox proportional hazards model to handle time-to-event data where the PH assumption is not met have been suggested and implemented [5–7]. These extensions often remain dependent on restrictive functions such as the heaviside functions that may be difficult to construct and implement or fail to fit the dataset in question. Other analysis approaches to handle non-proportional hazards include methods such as stratification, but these limit the ability to estimate the effect(s) of the stratification variable(s).

Survival trees and random survival forests (RSF) are an attractive alternative approach to the Cox proportional hazards models when the PH assumption is violated [8]. These methods are extensions of classification and regression trees and random forests (RF) [9, 10] for time-to-event data. Survival tree methods are fully non-parametric, flexible, and can easily handle high dimensional covariate data [11–13]. Drawbacks of random survival forests include the common drawbacks of random forests including a bias towards inclusion of variables with many split points [14–17]. This effect leads to a bias in resulting summary estimates such as variable importance [15, 17]. Conditional inference forests (CIF) are known to reduce this selection bias by separating the algorithm for selecting the best covariate to split on from that of the best split point search [15, 17, 18].

Despite the fact that the CIF survival model has been identified to reduce bias in covariate selection for splitting in survival forest models, no study has been done to compare the predictive performance of the CIF model and random survival forest models on time-to-event data in the presence of covariates that have many and fewer split-points. This study for the first time, examines and compares the predictive performance of the CIF and the two random survival forest models through a simulation study. Bootstrap cross-validated estimates of the integrated Brier scores were used as measures of predictive performance [19]. In total, twenty-two time-to-event datasets were simulated. Eighteen of the datasets were simulated in such a way that they either have binary covariates (few split-points), polytomous covariates (many split-points) or both. Four of the datasets were simulated in such a way that they have covariate interactions. Other properties of these datasets are further described in “Methods” section. The two real datasets used in this study are Dataset 1, which investigates the survival of 6692 children under the age of five in Uganda and contains categorical covariates with many levels (polytomous covariates). Dataset 2 evaluates the survival of 107 patients with extremely drug resistant tuberculosis (XDR TB) in South Africa. It is a small dataset and contains only categorical binary covariates.

This article is structured as follows: The “Methods” section describes the methods used. We discuss the methods used to evaluate the methods in the “Model evaluation” section. In the “Simulation study” section, we present the simulation study together with the simulation results. The “Real data application” section introduces the two real datasets that we used in this study and also gives the corresponding real data analyses results and lastly the “Discussion and conclusions” section presents the discussion and conclusions drawn from this study.

## Methods

A random survival forest (RSF) is an assemble of trees method for analysis of right censored time-to-event data and an extension of Brieman’s random forest method [14, 20]. Survival trees and forests are popular non-parametric alternatives to (semi) parametric models for time-to-event analysis. They offer great flexibility and can automatically detect certain types of interactions without the need to specify them beforehand [13]. A survival tree is built with the idea of partitioning the covariate space recursively to form groups of subjects who are similar according to the time-to-event outcome. Homogeneity at a node is achieved by minimizing a given impurity measure. The basic approach for building a survival tree is by using a binary split on a single predictor. For a categorical covariant *X*, a split is defined as *X*≤*c* where *c* is some constant. For a categorical covariate *X* with many split-points, the potential split is *X*∈{*c*_1_,…,*c*_*k*_} where *c*_1_,…,*c*_*k*_ are potential split values of a predictor variable *X*. The goal in survival tree building is to identify prognostic factors that are predictive of the time-to-event outcome. In tree building, a binary split is such that the two daughter nodes obtained from the parent node are dissimilar and several split-rules (different impurity measure) for time-to-event data have been suggested over the years [13, 21].

The impurity measure or the split-rule of the algorithm is very important in survival tree building. In this article, we used the log-rank and the log-rank score split-rules [22–24].

### The log-rank split-rule

Suppose a node *h* can be split into two daughter nodes *α* and *β*. The best split at a node *h*, on a covariate *x* at a split point *s* ^∗^ is the one that gives the largest log-rank statistic between the two daughter nodes [22]. The algorithm for building a survival tree using the split-rule based on the log-rank statistic [13, 22, 25, 26] is given in Algorithm 1 below.



### The log-rank score split-rule

The log-rank score split-rule [23] is a modification of the log-rank split-rule mentioned above. It uses the log-rank scores [24]. Given *r*=(*r*_1_,*r*_2_,…,*r*_*N*_), the rank vector of survival times with their indicator variable (*T*,*δ*)=((*T*_1_,*δ*_1_),(*T*_2_,*δ*_2_),…,(*T*_*N*_,*δ*_*N*_)) and that *a*=*a*(*T*,*δ*)=(*a*_1_(*r*),*a*_2_(*r*),…,*a*_*N*_(*r*)) denotes the score vector depending on ranks in vector *r*. Assume that the ranks order the predictor variables in such away that *x*_1_<*x*_2_<…<*x*_*N*_. The log-rank scores for an observation at *T*_*l*_ is given by:

1 $$ a_{l}=a_{l} \left(T,\delta\right)=\delta_{l} -\sum_{k=1}^{\gamma_{l}(T)}\frac{\delta_{k}}{N-\gamma_{k}(T)+1}\,{,}   $$

where

$$\gamma_{k}(T)=\sum_{l=1}^{N} \chi \{T_{l} \leqslant T_{k} \} $$

is the number of individuals that have had the event of interest or were censored before or at time *T*_*k*_.

2 $$ i\left(x,s^{\star}\right)=\frac{\sum_{x_{j}\leq s^{\star}}\left(a_{j}-R_{1}\bar{a}\right)}{\sqrt{R_{1}\left(1-\frac{R_{1}}{N}\right)S_{a}^{2}}}\,,  $$

where $\bar {a}$ and $S_{a}^{2}$ are the mean and sample variance of the scores {*a*_*j*_:*j*=1,2,…*n*}. The best split is the one that maximizes |*i*(*x*,*s* ^⋆^)| over all $x_{j}^{'}$s and possible splits *s* ^⋆^.

Trees are generally unstable and hence researchers have recommended the growing of a collection of trees [10, 27], commonly referred to as random survival forests [20, 26].

### Random survival forests algorithm

The random survival forests algorithm implementation is shown in Algorithm 2 [20, 26].



For this study, we used the log-rank and the log-rank score split-rules in Step 2 of the algorithm. Two random survival forest algorithms were generated denoted as RSF1 and RSF2. RSF1 consists of survival trees built using the log-rank split-rule whereas RSF2 consists of survival trees built using the log-rank score split-rule.

The random survival forests algorithm, has been criticised for having a bias towards selecting variables with many split points and the conditional inference forest algorithm has been identified as a method to reduce this selection bias. Conditional inference forests are formulated in such a way that it separates the algorithm for selecting the best splitting covariate is separated from the algorithm for selecting the best split point [15–18]. To illustrate this, consider a dataset with a time-to-event outcome variable *T* and two explanatory variables *x*_1_ and *x*_2_ with *k*_1_ and *k*_2_ possible split-points, respectively. Furthermore, consider that *T* is independent of *x*_1_ and *x*_2_, and that *k*_1_<*k*_2_. In the random survival forests algorithm, the search for the best covariate to split on and the best split-point by comparing the effect for both the covariates on *T*, gives *x*_2_ the highest probability of being selected just by chance.

### Conditional inference trees and forests

Algorithm 3 outlines the general algorithm for building a conditional inference tree as presented by [28].



For time-to-event data, the optimal split-variable in step 1 is obtained by testing the association of all the covariates to the time-to-event outcome using an appropriate linear rank test [28, 29]. The covariate with the strongest association to the time-to-event outcome based on permutation tests [28], is selected for splitting. In covariates selection, a linear rank test based on the log-rank transformation (log-rank scores) is performed. Using the distribution of the resulting rank statistic, *p*-values are evaluated and the covariates with minimum *p*-value is known to have the strongest association to the outcome [17, 30, 31]. Although the standard association test is done in the first step, a standard binary split is done in the second step. A single tree is considered unstable and hence research has recommended the growing of an entire forest [9, 10, 20]. The forest of conditional inference trees results into a conditional inference (CIF) model. The CIF model algorithm for time-to-event data is implemented in the R package called party.

To compare the performance of the three models used in this study, integrated Brier scores are used [32] which are described in the section below.

## Model evaluation

Brier scores [32] are used to compare the predictive performance of the two random survival forests models of all models. At a given time point *t*, the Brier score for a single subject is defined as the squared difference between observed event status (e.g., 1=alive at time *t* and 0=dead at time t) and a model based prediction of surviving time *t*. Using the test sample of size denoted as *N*_test_, Brier scores at time *t* are given by

3 $$ \begin{aligned} BS(t)&=\frac{1}{N_{\text{test}}}{\sum_{l=1}^{N_{\text{test}}}}\left\{ \left[0-\widehat{S}\left(t|x\right)\right]^{2}\frac{I\left(t_{l} \leq t,\delta_{l}=1\right)}{ \widehat{G}\left(t_{l}|x\right)}\right.\\ &\quad\left.+\left[1-\widehat{S}\left(t|x\right)\right]^{2}\frac{I\left(t_{l}>t\right)}{\widehat{G}\left(t|x\right)}\right\}\,. \end{aligned}  $$

Where $\widehat {G}\left (t|x\right)\approx P\left (C > t|X=x\right)$ is the Kaplan-Meier estimate of the conditional survival function of the censoring times.

The integrated Brier scores (*IBS*) are given by

$$\begin{array}{*{20}l} IBS &=\int_{0}^{\max(t)}BS(t)dt\,. \end{array} $$

To avoid the problem of overfitting that arises from using the same dataset to train and test the model, we used the Bootstrap cross-validated estimates of the integrated Brier scores [19]. The prediction errors are evaluated in each bootstrap sample.

These have been implemented in the pec package [19]. We fit a pec object with the three rival prediction models (RSF1, RSF2 and CIF). The three models were passed on as a list to the pec object and chose *s* *p* *l* *i* *t* *M* *e* *t* *h* *o* *d*=*B* *o* *o* *t*632*p* *l* *u* *s*. We set *B*=5, to have reasonable run times and reported Bootstrap cross-validated estimates for integrated Brier scores. Prediction error rates of 50% or higher are useless because they are no better than tossing a coin [12, 33].

## Simulation study

### Simulated time-to-event datasets

To simulate time-to-event datasets for this study, two frameworks were used. The first framework is more flexible and it generates time-to-event data from known distributions for proportional hazard models by inverting the cumulative baseline hazard function. The desired censoring parameters were achieved by randomly generating them from a binomial distribution. This framework was used to generate polytomous and datasets with covariate interactions [34–36]. The second framework uses a nested numerical integration and a root-finding algorithm to choose the censoring parameter that achieves predefined censoring rates in simulated time-to-event datasets [34]. This framework was used to generate time-to-event datasets with binary covariates.

#### Time-to-event datasets with binary covariates

Ten covariates are considered in each simulated dataset that is, *X*_*j*_={*X*_1_,*X*_2_,*X*_3_,*X*_4_,*X*_5_,*X*_6_,*X*_7_,*X*_8_,*X*_9_, *X*_10_}. Each of the covariates is randomly generated from a Bernoulli distribution with probability *p*_*j*_, *X*_*j*_∼*B*(*p*_*j*_). Weibull event times were considered, these were generated using a baseline hazard function $h_{0}(t)=\frac {\theta }{\rho ^{\theta }}t^{\theta -1}\,$. The scale parameter is given by $\lambda =\exp \left (-\frac {\beta _{0}}{\theta }-\sum _{j=1}^{n}\frac {\beta _{j}}{\theta }X_{j}\right)$, where *β*_0_ is the coefficient of the intercept term log(*ρ* ^−*θ*^). The shape parameter *θ* was set at 0.8,1.5, or 1 to represent decreasing, increasing and constant hazard, respectively. The corresponding intercept for each dataset was *β*_0_=−0.98,−1.44, or 0.9. The regression coefficients for the 10 covariates were defined as {0.5,−0.045,0.6,−0.03,−2,0.5,0.25,−0.04,0.33,0.3}, {0.1, −0.8,0.5,−0.2,−3,0.7,0.2,0.4,0.3}, {0.4,−0.7,1,−2,−3,0.7,0.06,0.5−0.43,0.3}, respectively. Censoring times were generated from a Weibull distribution with a shape parameters 0.4,2.4, or 1. The censoring parameter *θ* was computed numerically to get 50*%*,20*%* and 80%, respectively. In total, six time-to-event datasets were generated from this covariate design and other properties of these datasets are stated in Table 1.

**Table 1:** Simulated time-to-event datasets

Properties of simulated time-to-event datasets				
Type of covariates	Datasets	Sample size	% of censoring	Nature of the
				hazard
Binary	Data 1	100	80	Increasing
	Data 2	100	50	Decreasing
	Data 3	250	20	Constant
	Data 4	1000	80	Increasing
	Data 5	1500	50	Decreasing
	Data 6	2000	20	Constant
Polytomous	Data 1	100	20	Increasing
	Data 2	100	80	Constant
	Data 3	250	50	Decreasing
	Data 4	1000	20	Increasing
	Data 5	1500	80	Constant
	Data 6	2000	50	Decreasing
Binary & polytomous	Data 1	1000	20	Increasing
	Data 2	100	80	Decreasing
	Data 3	250	50	Constant
	Data 4	1000	20	Increasing
	Data 5	1500	80	Decreasing
	Data 6	2000	50	Constant
Interactions	Data 1	100	20	Increasing
	Data 2	100	50	Decreasing
	Data 3	1000	20	Increasing
	Data 4	1500	50	Decreasing

#### Time-to-event datasets with polytomous covariates

Covariates were generated by sampling with replacement from a list of desired categories. Event times were generated by inverting cumulative hazard function. *T*=−1∗ log(*U*)∗*ρ*∗*e* *x* *p*(−*λ*)^(^1/*θ*). Where $\lambda =\exp \left (-\frac {\beta _{0}}{\theta }-\sum _{j=1}^{n}\frac {\beta _{j}}{\theta }X_{j}\right)$. The shape parameter *θ* was set at 0.5,1.5 and 1 to yield time-to-event datasets with a decreasing, increasing and a constant hazard, respectively. The corresponding censoring times were also generated from a Weibull distribution with a shape and scale parameter of 0.4, 1.2 and 1.1, receptively. In this covariate design, six datasets were generated. Other properties for these datasets are given in Table 1.

#### Time-to-event datasets with binary and polytomous covariates

We used the same frame work for generating survival times as that of generating time-to-event datasets with polytomous covariates described above. Binary covariates, were added to the dataset by generating them from a Bernoulli distribution. In total, six datasets were generated.

#### Time-to-event datasets with covariate interactions

We used the same frame work for generating survival times as that for generating time-to-event datasets with polytomous covariates and four datasets were generated. The codes used to generate these datasets is provided as an additional file. In total, twenty-two datasets are generated for this simulation study. The Table 1 presents properties for each simulated dataset.

In this study, the two random survival forest methods, that is, the one consisting of trees built with the log-rank split-rule (RSF1) and the other consisting of survival trees built with the log-rank score split-rule (RSF2) are fit to the data and compared to the CIF model.

To have reasonable run times, 100 survival trees are grown for each survival forest fitted on each simulated dataset and this is repeated 100 times. The models are then evaluated using bootstrap cross-validated estimates of the integrated Brier scores. This estimate is recorded from each fitted survival forest [37–39]. All computations and analyses were carried out in R, using R version 3.3.2. We used the randomForestSRC [26], party [40], pec [19], rms [41], and doMC packages.

## Results on simulated datasets

For each repetition, bootstrapped-cross-validated integrated brier scores were recorded. The results are then reported using box-plots as shown below.

**Fig. 1:**
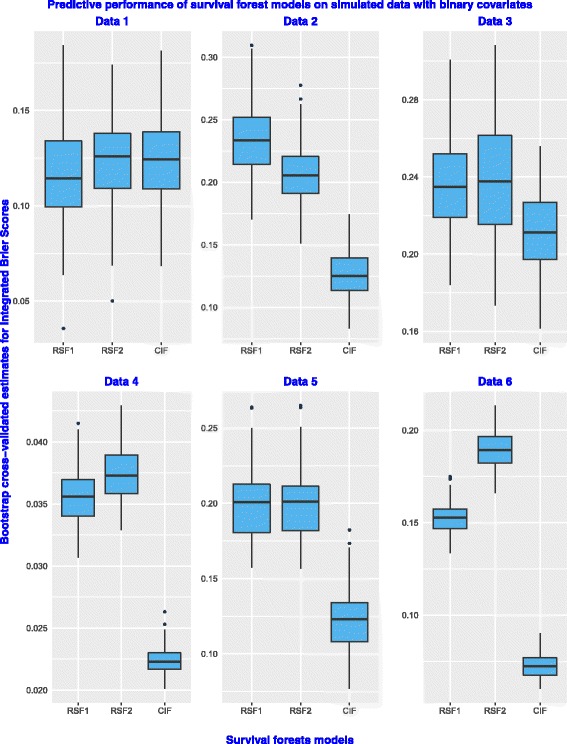
Predictive performance on simulated datasets with binary covariates

**Fig. 2:**
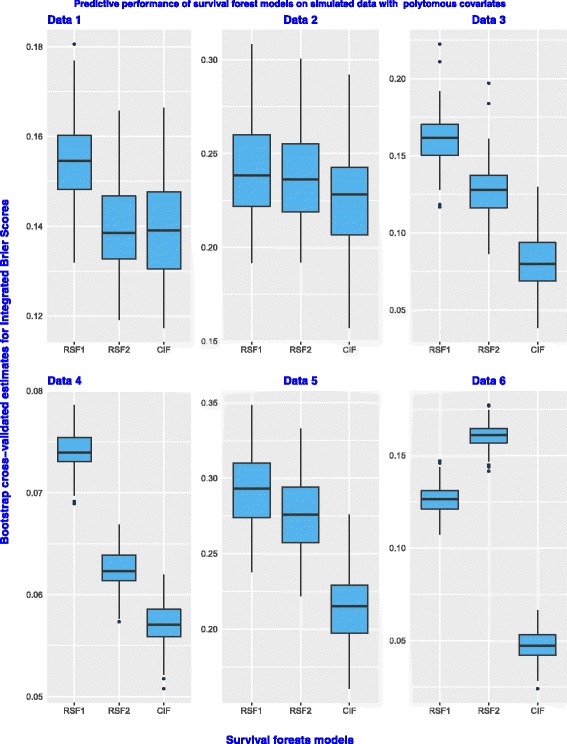
Predictive performance of the three survival forest models on simulated datasets with polytomous covariates

Figure 1 presents box plots of the prediction errors for RSF1, RSF2 and the CIF models on all the six datasets simulated with binary covariates. In general, all models have a good predictive performance on the dataset.This is because all the prediction error values are below the 50% cut-off point. However, there are some unique differences in the predictive performance between two random survival forests and the CIF model which can not be ignored. On Data 1, the prediction errors for the CIF model are sandwiched between the error values for RSF1 and RSF2. The prediction error values for The CIF model on the remaining five datasets are lowest compared to those of the RSF1 and RSF2 model. The box plots of the error values for the three models appear to be almost symmetrical. The results therefore indicate that the predictive performance for the two random survival forests and the conditional inference survival forest model is similar or comparable in performance on simulated time-to-event data with only binary covariates. Figure 2 indicates that all the three models have a good predictive performance on all the six datasets because the error values are below the 50% mark. Although the prediction error values for the CIF model appear to be at par with those of RSF2 on Data 1, the model has the lowest error values compared to RSF1 and RSF2 on the remaining five datasets. This is not a surprise because the CIF model is known to be superior in performance to random survival forests models in the presence of covariates with many split-points.

**Fig. 3:**
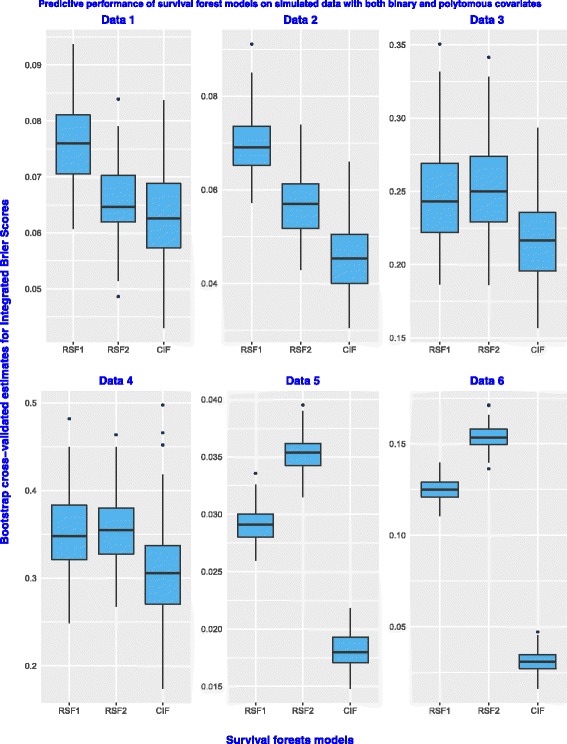
Predictive performance on simulated datasets with binary and polytomous covariates

The results presented by using box plots in Fig. 3 give prediction errors of RSF1, RSF2 and the CIF model on six datasets simulated to have both binary and polytomous covariates. On all the six datasets, the CIF model has the lowest prediction error rates. This is because conditional inference forests have an added advantage for prediction in the presence of covariates with many split-points because of the way it does the search for covariate selection and split-point.

**Fig. 4:**
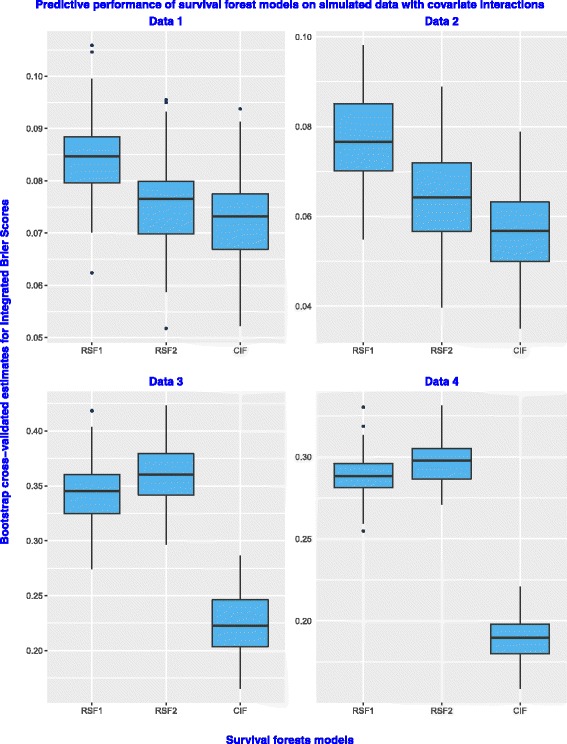
Predictive performance of the three survival forest models on simulated datasets with covariate interactions

Figure 4 presents box plots for predictive performance of the three survival forest model on simulated time-to-event data with covariate interactions. The prediction error values for the CIF model are lowest on all the six datasets. Since covariate interactions are simulated in such a way that some of the covariates have many split-points, the superiority in performance of the CIF model was not a surprise.

Generally, all the three survival forest models have a good predictive performance based on the bootstrap cross-validated estimates of integrated Brier score. However, there are some differences in the performance of each of the models on each of the simulated dataset as discuss above. The results in summary suggest that conditional inference forests have a good predictive performance compared to the two random survival forest models especially on time-to-event datasets with polytomous covariates. The model is comparable in predictive performance to random survival forests models in the analysis of simulated time-to-event datasets with binary covariates.

## Real data application

To further investigate the results obtained from the simulation study on the predictive performance of the three survival forest models, we analysed two real datasets whose covariate properties are similar to those used in the simulation study. Note that in identifying the most important covariates in explaining survival in the analysis of both datasets, permutation importance was used as measure of variable importance [20, 42].

### Dataset 1

The dataset can be found on the demographic health survey website [43]. In this survey, a representative sample of 10,086 households was selected during the 2011 Uganda Demographic and Health Survey (UDHS). The sample was selected in two stages. First a total of 404 enumeration areas (EAs) were selected from among a list of clusters sampled for the 2009/10 Uganda National Household Survey (2010 UNHS). In the second stage of sampling, households in each cluster were selected from a complete listing of households. Eligible women for the interview were aged between 15–49 years of age who were either usual residents or visitors present in the selected household on the night before the survey. Out of 9247 eligible women, 8674 were successively interviewed with a response rate of 94*%* (91*%* in urban and 95% in rural areas). The study population for this analysis includes infants born between exactly one and five years preceding the 2011 UDHS.

### Explanatory variables

In this dataset, 19 covariates are considered for analysis and their choice was based on literature studies [44–46]. To some extent, other limitations like high level of missingness in the dataset influenced our covariate choice. The dataset is readily available from the Demographic and Health Survey Data website [43]. Summary characteristics can be found in Table 2.

**Table 2:** Characteristics and the distribution of deaths for covariates in Dataset 1

Characteristics	Dead N(%)	Alive N(%)	Total	Characteristics	Dead N(%)	Alive N(%)	Total
Mother’s education level				Mother’s occupation			
Illiterate Mothers	344(7.7)	4149(92.3)	4493	Not-working	93(6.9)	1260(93.1)	1353
Mother completed primary	119(6.4)	1749(93.6)	1868	Sales and Services	110 (6.5)	1589 (93.5)	1699
Secondary and higher	14(4.2)	317(95.8)	331	Agriculture	274(7.5)	3366(92.5)	3640
Partner’s level of education				Births in past 5 years			
Illiterate Father	266(7.7)	3180(92.3)	3446	1-Birth	93(4.5)	1982(95.5)	2075
Father completed primary	170(6.9)	2287(93.1)	2457	2-Birth	227(6.5)	3288(93.5)	3515
Secondary and higher	41(5.2)	748(94.8)	789	3-Births	140(13.6)	887(86.4)	1027
Birth status				4-Births	17(22.7)	58(77.3)	75
Singleton births	431(6.7)	6048(93.3)	6479	Births in past 1 year			
Multiple births (Twins)	46(21.5)	167(78.5)	213	No-births	309(6.8)	4212(93.2)	4521
Sex of the child				1-Birth	163(7.6)	1971(92.4)	2134
Males	258(7.8)	3067(92.2)	3325	2-Births	5(13.5)	32(86.5)	37
Females	212(6.3)	3155(93.7)	3367	Children Under 5 in Household			
Type of place of residence				No-child	101(34.9)	188(65.1)	289
Urban	81(5.8)	1308(94.2)	1389	1-Child	178(10.5)	1511(89.5)	1689
Rural	396(7.5)	4907(92.5)	5303	2-Children	146(4.9)	2831(95.1)	2977
Wealth index				3-Children	35(2.5)	1349(97.5)	1384
Poorest	131(7.5)	1623(92.5)	1754	4-Children	17(4.8)	336(95.2)	353
Poorer	112(8.5)	1205(91.5)	1317	Mother’s age group			
Middle	86(7.2)	1109(92.8)	1195	Less than 20 years	29(8.9)	296(91.1)	325
Richer	72(6.9)	969(93.1)	1041	20-29 years	235(6.5)	3376(93.5)	3611
Richest	76(5.5)	1309(94.5)	1385	30-39 years	164(7.4)	2054(92.6)	2218
Children ever born				40 years ^+^	49(7.9)	489(90.1)	538
One child	20(3.3)	581(96.7)	601	Birth order number			
Two children	81(7.1)	1065(92.9)	1146	First child	95(7.6)	1154(92.4)	1249
Three children	67(6.6)	953(93.4)	1020	Second to Third child	117(5.6)	1974(94.4)	2091
Four and more	309(7.9)	3616(92.1)	3925	4^*th*^-6^*th*^ child	149(7.1)	1949(92.9)	2098
Birth order number				^*th*^+child	116(9.3)	1138(90.7)	1254
First child	95(7.6)	1154(92.4)	1249	Sex of household head			
Second to Third child	117(5.6)	1974(94.4)	2091	Male	341(6.7)	4771(93.3)	5112
4^*th*^-6^*th*^ child	149(7.1)	1949(92.9)	2098	Female	136(8.6)	1444(91.4)	1580
^*th*^+child	116(9.2)	1138(90.8)	1254	Source of drinking water			
Religion				Piped water	76(5.9)	1204(94.1)	1280
Catholics	217(7.4)	2722(92.6)	2939	Borehole	216(7.3)	2731(92.7)	2947
Muslims	69(7.5)	852(92.5)	921	Well	93(6.9)	1261(93.1)	1354
Other Christians	187(6.8)	2571(93.2)	2758	Surface/Rain/Pond/Lake/tank	70(8.5)	756(91.5)	826
Others	4(5.4)	70(94.6)	74	Other	22(7.7)	263(92.3)	285
Type of toilet facility				Age at first birth			
Flush toilet	5(4.1)	116(95.9)	121	Less than 20 years	347(7.5)	4291(92.5)	4638
Pitlatrine	376(6.9)	5031(93.1)	5407	20-29 years	127(6.3)	1899(93.7)	2026
No-facility	96(8.2)	1068(91.8)	1164	30-39 years	3(12.0)	22(88.0)	25

The time-to-event outcome of interest is time to death of children under the age of five. The range of values of this outcome lie between one month and 59 months of age. Children that were alive at the time of the interview were considered to be right censored. The dataset has a high censoring rate of 93%.

### Dataset 2

Between August 2002 and October 2012, a total of 107 adult patients with microbiologically confirmed XDR-TB from three provinces in South Africa, were hospitalised for treatment in three tuberculosis treatment facilities (Brooklyn Chest Hospital, Western Cape [B], Gordonia Hospital, Upington, Northern Cape, [H] and Sizwe Tropical Disease Hospital, Johannesburg, Gauteng [S]). All the three hospitals are specialist referral centres for the treatment of drug resistant TB, aimed at serving patients from across respective provinces. This dataset has been published in [47].

#### Explanatory variable

The covariates of interests were selected based on literature [47, 48] and limitations of high level of missingness.

Table 3 shows the distribution of deaths in each of the covariates considered. The outcome variable is survival time in days from diagnosis of XDR-TB.

**Table 3:** Characteristics and the distribution of deaths for covariates in Dataset 2

Characteristics	Dead N(%)	AliveN (%)	Total	Characteristics	Dead N(%)	Alive N(%)	Total
Age at diagnosis				Ethionamide			
Below 30	35(81.3)	8(18.6)	43	Not prescribed	25(64.10)	14(35.89)	39
Above 30	43(68.25)	20(31.75)	63	Prescribed	54(79.41)	14(20.59)	68
Gender				Ofloxacin			
Females	41(83.67)	8(16.33)	49	Not prescribed	48(70.59)	20(29.41)	68
Males	38(65.52)	20(34.48)	58	Prescribed	31(79.49)	8(20.51)	39
smoking status				Ofloxacin and moxifloxacin			
No	28(65.12)	15(34.88)	43	Not prescribed	72(72.73)	27(27.27)	99
Yes	38(79.17)	10(20.83)	48	Prescribed	7(87.50)	1(12.50)	8
HIV plus ART status				Amikacin			
HIV -ve	46(73.02)	17(26.98)	63	Not prescribed	76(73.79)	27(26.21)	103
HIV +ve ART	24(68.57)	11(31.43)	35	Prescribed	3(75.00)	1(25.00)	4
HIV +ve no ART	9 (100.00)	0 (0.00)	9	Capreomycin			
Cohort				Not prescribed	8(88.98)	1(11.11)	9
B	54(83.08)	11(16.92)	65	Prescribed	71(72.45)	27(27.55)	98
N	12(80.00)	3(20.00)	15	Dapsone			
S	13(48.15)	14(51.85)	27	Not prescribed	43(67.19)	21(32.81)	64
Race				Prescribed	36(83.72)	7(16.28)	43
Blacks	34(64.15)	19(35.85)	53	Augmentin			
Mixed ancestry	45(83.33)	9(16.67)	54	Not prescribed	28(66.67)	14(33.33)	42
Drugs used				Prescribed	51(78.46)	14(21.54)	65
Isoniazid				Clofazamine			
Not prescribed	57(83.82)	11(16.18)	68	Not prescribed	70(82.35)	15(17.65)	85
Prescribed	22(56.41)	17(43.59)	39	Prescribed	9(40.91)	13(59.09)	22
Etambutol				Azithromycin			
Not prescribed	39(66.10)	20(33.89)	59	Not prescribed	75(76.53)	23(23.47)	98
Prescribed	40(83.33)	8(16.67)	48	Prescribed	4(44.44)	5(55.56)	9
Pyrazinainamide				Amoxicillin			
Not prescribed	14(58.33)	10(41.67)	24	Not prescribed	49(71.01)	20(28.99)	69
Prescribed	65(78.3)	18(21.69)	83	Prescribed	30(78.95)	8(21.05)	38
				Clarithromycin			
				Not prescribed	19(70.37)	8(29.63)	27
				Prescribed	60(75.00)	20(25.00)	80

The median survival time was 30.9(IQR =19.5) months. A total of 79 (74%) patients died, with a low censoring rate of 26%.

### Analysis

#### Dataset 1

**Fig. 5:**
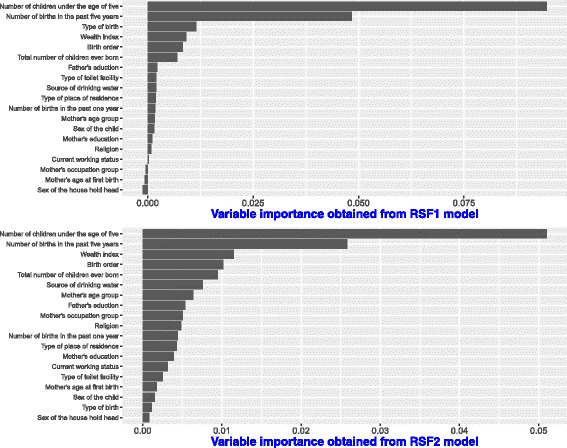
Variable importance scores obtained from RSF1 and RSF2 model on Dataset 1

Results from the two random survival forest models applied to Dataset 1 shown in Fig. 5 identify the number of children under the age of five in the household as the most informative predictor of time to death for children under-five in Uganda.

Other covariates strongly associated to under-five child mortality in Uganda include; the number of births in the past five years, birth order, wealth index and the total number of children ever born. Both random survival forest models have similar results in identifying the same factors affecting the time to survival of children under the age of five years in Uganda.

**Fig. 6:**
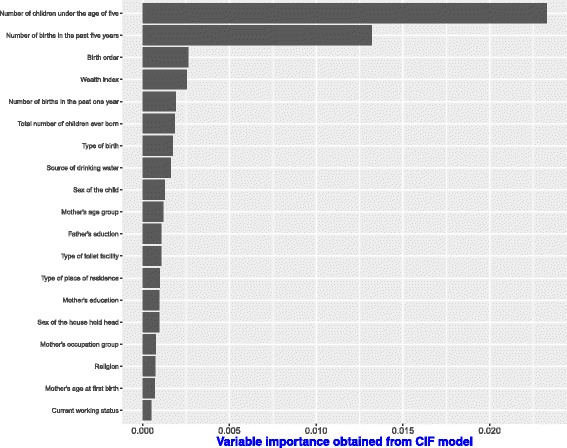
Variable importance scores obtained under CIF model on Dataset 1

The results from the CIF model on the same dataset in Fig. 6, agree with the top two predictors by RSF1 and RSF2. The top predictors are; number of children under the age of five in a household and the number of births in the past five years. Some covariates in the CIF model that move up in ranks compared to RSF1 and RSF2 include; the number of births in the past one year and the sex of the household head. These two covariates were also found important in explaining under-five child mortality rates by [49] using the Cox proportional hazards models.

#### Dataset 2

**Fig. 7:**
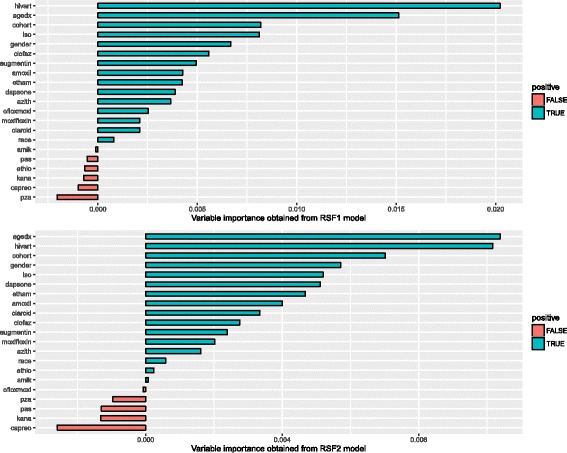
Variable importance obtained from fitting RSF1 and RSF2 model on Dataset 2

Figure 7, presents results of variable importance from RSF1 and RSF2 on Dataset 2. The covariates are ranked according to their degree of importance in RSF1. The combined HIV/ART status is ranked most important among the covariates considered in predicting the time to death of patients with XDR TB in both random survival forest models. Age at diagnosis and specific prescribed drugs (Isoniazid, Amoxicillin and Clofazamine) are also ranked highly important. The same drugs were found to be predictive in the multivariate Cox analysis [47].

**Fig. 8:**
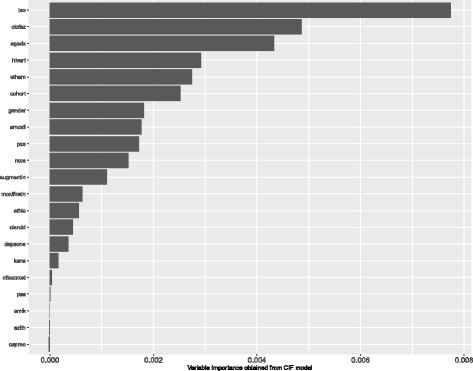
Variable importance obtained under CIF model on Dataset 2

The results obtained from fitting the CIF model on the same dataset in Fig. 8, indicate that the age at diagnosis and HIV/ART status are again highly associated with the outcome.

The three survival forest models give similar results in determining the factors affecting the survival of patients with XDR TB.

## Results on real datasets

**Fig. 9:**
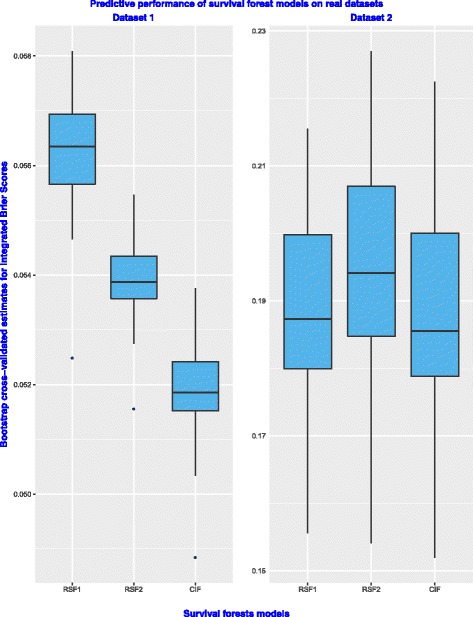
The predictive performance of the two random survival forest models and the conditional inference forest model on Dataset 1 and 2

For each survival forests, 100 trees were built and this was repeated 50 times. For each repetition, bootstrapped-cross-validated integrated brier scores were recorded. The results on predictive performance of all the models used in this study on Dataset 1 and Dataset 2 is shown in Fig. 9. Overall, the three models show a good predictive performance on the two real datasets as shown in Fig. 9. On Dataset 1, the conditional inference forest model has the lowest prediction error values compared to the two random survival forest models. On Dataset 2, however, the two random survival forest model are at par in predictive performance compared to the conditional inference forests model. Infact the prediction error values of the three models are all positively skewed. These results on the predictive performance of the three survival forest models confirm that the CIF model is superior in predictive performance to the two random survival forest models on the real survival dataset with covariates that have many split-points. The study also shows that the CIF model and the two random survival forests are comparable in predictive performance on real time-to-event datasets with covariates that have fewer split-points. Similar results were obtained from the simulation study.

## Discussion and conclusions

In this study, we compared the predictive performance of three survival forests models on twenty-two simulated time-to-event datasets and two real time-to-event datasets. First, eighteen datasets were simulated to have covariate properties of interest that is, fewer split-points vs many split-points. Four more time-to-event datasets with covariate interactions were also simulated. The first two forest models are random survival forests models with trees built based on the log-rank and the log-rank score split-rule, respectively. The third survival forest model consists of conditional inference trees.

The results from comparing the predictive performance on three survival forest models on simulated time-to-event datasets indicate that the three random survival forest models have a good predictive performance. Despite this fact, the study has shown that there are some variations in predictive performance for these three models in the presence of covariates with many vs those with fewer split-points. The study suggests that conditional inference forests are superior in predictive performance to random survival forests on time-to-event datasets with polytomous covariates. The results also indicate that the three models are comparable in predictive performance on time-to-event datasets with categorical covariates that are binary in nature. The superiority in performance of the CIF model is likely due to the way it handles the split variable and the split point selection especially in the presence of covariates with many split-points. These results are similar to those from the simulation study. This study therefore confirms the results that conditional inference forests are desirable in analysing time-to-event data consisting of covariates with many split-points. This results is therefore in agreement with the assertion made from a study by [28] that the CIF model is desirable in analysing time-to-event data in the presence of covariates with many split points.

The main finding of this study is that random survival forests perform comparably to conditional inference forests in analysing time-to-event data consisting of covariates with few split-points and that conditional inference forests are desirable in situations where the data consists of covariates with many split-points. It is therefore important for researchers to select the best survival forest model to analyse any time-to-event dataset based on the nature of its covariates.

Note that the conditional inference forest for time-to-event analysis and random survival forests have a difference in the way they calculate the predicted time-to-event probabilities and it is not yet clear whether this has an influence on their overall predictive performance [19]. The CIF model utilizes a weighted Kaplan-Meier estimate based on all subjects from the training dataset and it therefore put more weight on terminal nodes where there is a large number of subjects at risk whereas random survival forests use equal weights on all terminal nodes. Further studies need to be done to understand whether this property has an influence on the predictive performance of these models.

The limitation of this study is that we used random survival forest models that consists of survival trees based on the log-rank split-rule. Recent studies have raised concerns that since the log-rank split-rule is based on the proportional hazards assumption, it may negatively affect the predictive performance of the survival forest model. A recent study has recommended the use of the integrated absolute difference between the two daughter nodes survival functions as the splitting rule especially in circumstances when the hazard functions cross [50]. Further studies would therefore compare the predictive performance of the CIF model to robust random survival forest models resulting from using robust split-rules especially in the presence of covariates that violate the PH assumption. Another limitation of the study is that the real datasets used had missing data and we assumed that the data was missing completely at random. Only a complete case analysis was considered which negatively affect outcomes.
